# Transcriptional Regulation of Endogenous Retroviruses and Their Misregulation in Human Diseases

**DOI:** 10.3390/ijms231710112

**Published:** 2022-09-04

**Authors:** Qian Zhang, Juan Pan, Yusheng Cong, Jian Mao

**Affiliations:** Key Laboratory of Aging and Cancer Biology of Zhejiang Province, Hangzhou Normal University School of Basic Medical Sciences, Hangzhou 311121, China

**Keywords:** endogenous retroviruses (ERVs), transcriptional regulation, cancer, neurodegenerative diseases

## Abstract

Endogenous retroviruses (ERVs), deriving from exogenous retroviral infections of germ line cells occurred millions of years ago, represent ~8% of human genome. Most ERVs are highly inactivated because of the accumulation of mutations, insertions, deletions, and/or truncations. However, it is becoming increasingly apparent that ERVs influence host biology through genetic and epigenetic mechanisms under particular physiological and pathological conditions, which provide both beneficial and deleterious effects for the host. For instance, certain ERVs expression is essential for human embryonic development. Whereas abnormal activation of ERVs was found to be involved in numbers of human diseases, such as cancer and neurodegenerative diseases. Therefore, understanding the mechanisms of regulation of ERVs would provide insights into the role of ERVs in health and diseases. Here, we provide an overview of mechanisms of transcriptional regulation of ERVs and their dysregulation in human diseases.

## 1. Introduction

**Figure 1:**
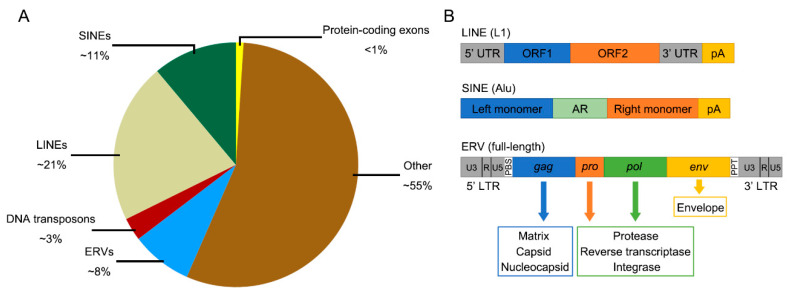
Organization and structure of transposable elements (TEs). (**A**) Pie chart shows the proportion of various selected genomic features within the human genome. (**B**) Genomic structures of LINE, SINE, and ERV. The general structure of a full-length ERV is shown. AR, adenine (A)-rich region. pA, poly (A) tail. PBS, primer binding site. PPT, polypurine tract.

Transposable elements (TEs) are repetitive genetic sequences that once had or still have the ability to transpose, that is, to mobilize and insert elsewhere in the genome [1]. Nearly half of the human genome consists of TEs [2,3] (Figure 1A). TEs can be categorized into two classes: elements that can be transposed via a DNA intermediate and a cut-and-paste mechanism (transposons), and those using an RNA and a copy-paste mechanism (retrotransposons) [4]. Retrotransposons are further divided into long terminal repeat (LTR) elements and more primitive and ancient non-LTR elements with an obligate intracellular life cycle [5]. Non-LTR retrotransposons consist of two main groups: long interspersed nuclear elements (LINEs), which encode their own proteins necessary for retrotransposition; and short interspersed nuclear elements (SINEs), which are short, noncoding RNAs that hijack the LINE protein machinery [5] (Figure 1A,B). Retrotransposons flanked by LTRs that have high similarities to exogenous retroviruses are termed endogenous retroviruses (ERVs), which are the remnants of ancient exogenous retroviral infections [6] (Figure 1B). These endogenized forms of viral sequences were derived from exogenous retroviral infections and integrations for germ cells and transmitted vertically through Mendelian inheritance [7]. In human, ERVs account for ~8% of the human genome [2] (Figure 1A). The complete genomic structure of ERVs is composed of *gag*, *pro*, *pol*, and *env*, flanked by two LTRs (Figure 1B). Among them, *gag* encodes for capsid, nucleocapsid, and matrix protein; *pro* encodes for protease; *pol* encodes for reverse transcriptase and integrase; and *env* encodes for envelope protein [7]. LTRs are non-coding regions that contain many regulatory functions (promoter, enhancer, polyA signal, and others) [7]. However, most of ERVs are non-protein coding due to the accumulation of mutations, insertions, deletions, and truncations [8,9]. Based on the sequence similarity of their *pol* regions with reverse transcriptase sequences of exogenous retroviruses, ERVs are divided into three main classes: class I (Gamma- and Epsilonretrovirus-like), class II (Alpha-, Beta-, Deltaretrovirus-, and Lentivirus-like), and class III (Spumaretrovirus-like) [10]. However, no Alpha-, Deltaretrovirus-, or Lentivirus-like elements were detectable in human genome [9]. The major classes of ERVs are shown in Table 1. Human ERVs (HERVs) are further classified into several groups based on the tRNA binding to the viral primer binding site (PBS) to prime reverse transcription. For example, HERV-K implies a group of proviruses using a lysine (K) tRNA as primer [11]. In some cases, the PBS sequence was unclear when novel elements were discovered, resulting in their names based on neighboring genes (e.g., HERV-ADP), clone number (e.g., HERV-S71), or amino acid motifs (e.g., HERV-FRD) [12]. Recently, a unified nomenclature system for ERVs that provides the ERV group, the genomic loci, and species was proposed, which can aid genome annotation and research of ERVs [12].

ERVs have been considered as “junk DNA sequences” for a long time [5]. However, it is becoming increasingly apparent that ERVs influence host biology through genetic and epigenetic mechanisms under particular physiological and pathological conditions, which provides both beneficial and deleterious effects for the host. For example, certain ERVs expression is essential for human embryonic development, whereas abnormal activation of ERVs is involved in numbers of human diseases, such as cancer and neurodegenerative diseases. Therefore, ERVs are under strict epigenetic regulation by the host, among which the methylation modifications of histone and DNA play significant roles. In this review, we will summarize recent findings on the mechanisms of transcriptional regulation of ERVs and their transcriptional dysregulation in human diseases.

## 2. Silencing and Transcriptional Regulation of ERVs

Currently, most discoveries about ERVs silencing and transcriptional regulation have been studied in mice, especially during embryonic development and in germ cells. Mouse embryonic stem cells (mESCs) are usually used as a cellular model to study the transcriptional regulation of ERVs, as the pluripotent state is capable of suppressing both exogenous and endogenous retroviruses [13,14,15]. Many mechanisms and concepts of transcriptional regulation of mouse ERVs may be applicable to human ERVs, but there may be discrepancies between them.

### 2.1. KRAB-ZFPs/TRIM28 Pathway Is a Master Regulator for ERVs Silencing

**Figure 2:**
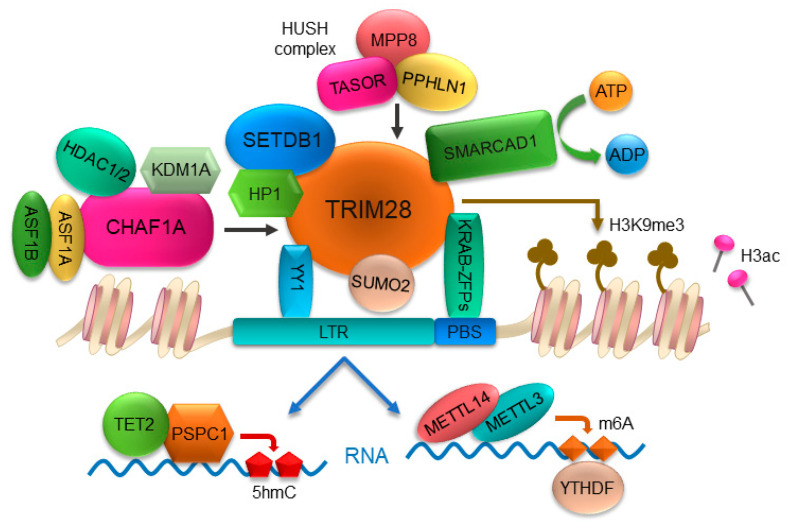
An overview model of ERVs silencing. ERVs are predominantly silenced by H3K9me3 through the canonical KRAB-ZFPs/TRIM28 pathway. KRAB-ZFPs bind to PBS region of ERVs and recruit TRIM28. Sumoylation of TRIM28 by SUMO2 enhances the recruitment of TRIM28 to ERVs. The ATPase activity of chromatin remodeler SMARCAD1 contributes to the occupancy of TRIM28 at ERVs. TRIM28 provides a scaffolding platform allowing for the recruitment of SETDB1, HP1, and HUSH complex, and the formation of macromolecular ensembles, which establish H3K9me3 in ERVs. Histone chaperone CHAF1A interacts with HP1, SETDB1, KDM1A, and HDAC1/2, modifying proviral chromatin with the repressive histone mark H3K9me3 and reducing the acquisition of active H3K4me3 and H3Ac marks. Histone chaperone isoforms ASF1A and ASF1B promote the localization of CHAF1A to ERVs. In addition to transcription-based silencing, RNA-mediated regulation of ERVs, such as epigenetic modifications of ERV RNAs, also play a critical role in silencing of ERVs.

A common target for ERVs silencing is the PBS, an essential sequence used to prime reverse transcription by a host tRNA as primer. The first example for this mechanism is ZFP809, a member of the family of Krüppel-associated box-containing zinc-finger proteins (KRAB-ZFPs), which binds the PBS^Pro^ of provirus [16]. Interestingly, the DNA-binding specificity of ZFP809 is evolutionarily conserved and predates the endogenization of retroviruses presently targeted by ZFP809 in *Mus musculus* [17]. ZFP809 contains two domains, a KRAB box at the N terminus that is responsible for the interaction with TRIM28 and a zinc-finger domain containing seven zinc fingers that provide its sequence-specific DNA-binding activity [18]. Besides ZFP809, additional KRAB-ZFPs have also been identified to bind ERVs sequences, through which mediate provirus silencing, including ZFP708, ZNF91/93, ZFP819, ZFP932, Gm15446, and YY1 [19,20,21,22,23]. The KRAB domain of KRAB-ZFPs mediates the recruitment of TRIM28, the master regulator of ERVs silencing [6,24]. TRIM28 (also known as KAP1, TIF1β, or KRIP-1), which was identified to bind to the KRAB domain of KRAB-ZFPs, functions as a scaffold for other repressive histone-modifying and -binding factors, including the histone methyltransferase SETDB1, the human silencing hub (HUSH) complex, and heterochromatin protein 1 (HP1), which catalyze heterochromatin formation and transcriptional repression [25] (Figure 2). *Trim28* is expressed in a variety of cell types with especially high levels during early embryonic development, in brain and mESCs [24,26,27]. Knockout of *Trim28* results in embryonic lethal at E8.5, highlighting its essential role in early development [26].

SETDB1 (also known as ESET or KMT1E) is a protein lysine methyltransferase methylating histone H3 at lysine 9 (H3K9) [28]. Unlike other H3K9 methyltransferases, SETDB1 and SETDB1-mediated H3K9me3 play critical roles for silencing of ERVs [29]. SETDB1 is mainly localized in cytoplasm [30] while ATF7IP promotes its nuclear import and inhibits its nuclear export [31]. SETDB1 interacts with TRIM28 and is recruited to ERVs by KRAB-ZFPs/TRIM28 pathway, then establishes H3K9me3 in ERVs [29] (Figure 2). Actually, the KRAB-ZFPs/TRIM28 pathway is central for the de novo recruitment of SETDB1 to ERVs [28]. In addition to SETDB1, several other histone methyltransferases have also been described for ERVs silencing, including SUV39H1, SUV39H2, G9a (also known as EHMT2), GLP (also known as EHMT1), and NSD2 [32,33,34,35]. Another well-known interaction partner of TRIM28 is the human silencing hub (HUSH) complex, comprising TASOR (also known as FAM208A), MPP8 (MPHOSPH8), and PPHLN1 (Periphilin 1) [36]. The HUSH complex is recruited to genomic loci rich in H3K9me3 and interacts with SETDB1 and MORC2, mainly repressing evolutionarily young retrotransposons, such as young L1 [36]. Furthermore, epigenetic silencing by the HUSH complex also mediates position-effect variegation in human cells [37]. Recently, a study described a functional connection between the mouse-orthologous “nuclear exosome targeting” (NEXT) and HUSH complexes, involved in nuclear RNA decay and the epigenetic silencing of TEs, respectively, suggesting that transcriptional and post-transcriptional machineries synergize to suppress the genotoxic potential of TE RNAs [38]. H3K9me3 reader proteins, such as HP1, can bind to pre-existing H3K9me3 and bridge with SETDB1 through direct interaction [39]. Despite the reported function of HP1 proteins in H3K9me-dependent gene repression and the critical role of H3K9me3 in transcriptional silencing of ERVs, the depletion of all three HP1 isoforms (HP1α, HP1β, and HP1γ) in mESCs is not sufficient for the derepression of selected ERVs [40]. This surprising finding is attributed that H3K9me3 may repress ERVs transcription via inhibiting deposition of covalent histone modifications required for transcription [40]. Regardless, additional studies aimed at characterizing the functional significance of H3K9 readers are clearly warranted.

### 2.2. Chromatin Remodeler and Histone Chaperone Maintain ERVs Silencing through KRAB-ZFPs/TRIM28 Pathway

The chromatin remodelers and the histone chaperones differing from well-known RNA chaperones or Janus chaperones have been considered as two important classes of factors involved in transcriptional regulation of ERVs, which are dependent on the KRAB-ZFPs/TRIM28 pathway. Recently, the SWI/SNF-like remodeler SMARCAD1 was identified as a key factor in the control of ERVs in mESCs [41]. As key regulators of nucleosome positioning, the SWI/SNF family of chromatin-remodeling complexes use energy generated through hydrolysis of ATP to slide or eject nucleosomes and promote chromatin access by moving nucleosomes, by which either activates or represses transcription [42,43]. For the transcriptional regulation of ERVs, SMARCAD1 is enriched at ERVs subfamilies class I and II, particularly at active IAPs, where it preserves repressive histone methylation marks. Importantly, recruitment of SMARCAD1 to ERVs is dependent on TRIM28 and the ATPase function of SMARCAD1 is required for SMARCAD1 and TRIM28 occupancy at ERVs (Figure 2), highlighting a critical role for SWI/SNF-like chromatin-remodeling activities in the establishment of ERVs silencing in mammals [41].

The association of histones with specific chaperone complexes is important for their folding, oligomerization, post-translational modification, nuclear import, stability, assembly, and genomic localization, which affects all chromosomal processes, including gene expression, chromosome segregation, and genome replication and repair [44]. Recently, a systematic genome-wide siRNA screen identified CHAF1A, a histone chaperone that assembles histones H3/H4 during DNA replication and repair [45,46], as a significant factor for silencing of ERVs [39]. It is shown that CHAF1A interacts with HP1, SETDB1, KDM1A, and HDAC1/2 [39,47,48] (Figure 2), modifying proviral chromatin with the repressive histone mark H3K9me3 and reducing the acquisition of active H3K4me3 and H3Ac marks [39]. ASF1 is also a chaperone that forms a complex with histones H3 and H4 [49]. The nucleosome assembly function of the two ASF1 isoforms, ASF1A and ASF1B, is shown to be responsible for localizing CHAF1A to proviral sequences [39].

ATRX is a chromatin remodeler and interacts with DAXX to form a histone chaperone complex, which deposits histone variant H3.3 into repetitive heterochromatin, including regions of retrotransposons, pericentric heterochromatin, and telomeres [50]. A series of studies revealed that ATRX and DAXX play roles for heterochromatin formation on ERVs through deposition of histone H3.3 [51,52,53]. The histone variant H3.3 belongs to the replication-independent class of variants and associated to both active chromatin states (e.g., H3K4me and H3K27ac) and heterochromatin states (e.g., H3K9me3 and H3K27me3) [54]. In mESCs, a study reported that recruitment of DAXX, H3.3 and TRIM28 to ERVs is co-dependent and occurs upstream of SETDB1, and H3.3 deletion leads to reduced H3K9me3 at ERVs regions and derepression of IAPs, establishing an important role for H3.3 in control of ERVs transcription in mESCs [53]. 

### 2.3. Sumoylation of TRIM28 Contributes to ERVs Silencing

Post-translational modification with small ubiquitin-related modifier (SUMO) proteins is one of the key regulatory protein modifications in eukaryotic cells. Hundreds of proteins involved in processes, such as chromatin organization, transcription, DNA repair, macromolecular assembly, protein homeostasis, trafficking, and signal transduction, are subject to reversible sumoylation [55]. Recent studies have shown that H3K9me3 deposition requires protein sumoylation, suggesting that the SUMO pathway functions as an important module in gene silencing and heterochromatin formation [56]. Importantly, the genome-wide screen for provirus silencing factors further confirmed the significant role of sumoylation for ERVs repression [39]. The SUMO family in mammals consists of four members: SUMO1, SUMO2, SUMO3, and SUMO4 [55]. Among them, SUMO2 orchestrates viral silencing through sumoylation modification of TRIM28 [39]. Sumoylation enhances the recruitment of TRIM28 to the proviral DNA, which in turn results in the modification of proviral chromatin with repressive histone H3K9me3 marks [39] (Figure 2). Nonetheless, further studies are needed to determine the mechanism of sumoylation in transcriptional regulation of ERVs.

### 2.4. DNA Methylation in ERVs Silencing

In addition to histone-based silencing, ERVs exhibit distinctive DNA methylation patterns [6]. Interestingly, KRAB-ZFPs/TRIM28 and SETDB1 are necessary to target ERV-containing loci for rapid de novo DNA methylation [57]. Three DNA methyltransferases in mammals (DNMT1, DNMT3A, and DNMT3B) have been intensively studied. The roles of DNA methylation in ERVs silencing appears to be cell type dependent [24,29,58,59]. For example, knockout of all three DNA methyltransferases in mESCs showed a complete loss of DNA methylation on ERVs, but only subtle derepression of ERVs was observed [29,60,61]. However, loss of DNA methylation activates ERVs expression in differentiated or somatic cells, such as mouse embryonic fibroblasts (MEFs) [58,59], while deletion of *Trim28* or *Setdb1* in MEFs does not lead to significant activation of ERVs [24,29]. Interestingly, deletion of *Trim28* or *Setdb1* in neural progenitor cells or pro-B cells results in strong ERVs derepression with only a slight reduction in DNA methylation [62,63,64,65]. These data indicate that the KRAB-ZFPs/TRIM28 pathway is primarily used for ERVs silencing in cells with stemness, whereas differentiated cells primarily rely on DNA methylation to suppress ERVs. As a member of DNMT3 family, DNMT3L (DNMT3-like) has no DNA methyltransferase activity but is capable of interacting with both DNMT3A and DNMT3B to stimulate their enzymatic activities [66]. Deletion of *Dnmt3l* in mouse testis prevents the de novo methylation of both LTR and non-LTR retrotransposons, leading to the activation of IAPs and L1, as well as meiotic failure [67]. Notably, a recent study reported a correlation between the silencing mechanism and the evolutionary age of ERVs [68]. Young LTRs tend to be CpG rich and are mainly suppressed by DNA methylation, while intermediate age LTRs are associated predominantly with histone modifications, particularly H3K9 methylation [68].

### 2.5. RNA-Mediated Regulation of ERVs

In addition to DNA-specific binding by co-repressors, histone chaperones, and chromatin remodelers, RNA-mediated targeting of ERVs also play a significant role for the silencing and transcriptional regulation of ERVs. It has been reported that siRNA- or antisense transcripts-based silencing pathways suppress IAPs and non-LTR retrotransposons such as L1 [69,70]. The most representative RNA-dependent gene silencing is *Xist,* the master regulator of X chromosome inactivation in mammals [71]. SPEN is a key factor for establishment of *Xist*-mediated silencing through directly recruited to *Xist* RNA [71]. A recent study showed that SPEN binds to retroviral RNA and performs a surveillance role to recruit chromatin-silencing machinery to these parasitic loci, suggesting that *Xist* may coopt ERVs RNA–protein interactions to repurpose powerful antiviral chromatin-silencing machinery [72]. Another mechanism for RNA-mediated transcriptional regulation of ERVs is the Piwi-interacting RNA (piRNA) pathway. piRNAs are a class of small RNAs that are 24–31 nucleotides in length and associate with PIWI proteins to form effector complexes known as piRNA-induced silencing complexes, which repress retrotransposons via transcriptional or post-transcriptional mechanisms [73]. It is in *Drosophila* that piRNA was first found to induce silencing through H3K9me3 formation [74]. In mice, depletion of Piwi proteins leads to derepression of IAPs and L1 [75,76].

It is worth noting that RNA epigenetic modifications play a significant role in regulation of ERVs. TET2, a member of the Ten-eleven translocation (TET) family, can be recruited to actively transcribed MuERVL RNAs by the RNA-binding protein PSPC1, then catalyzes 5hmC modification of MuERVL RNAs, resulting in their destabilization (Figure 2), which provides evidence for a functional role of transcriptionally active ERVs as specific docking sites for RNA epigenetic modulation [77]. m6A RNA methylation, which is catalyzed by the complex of methyltransferase-like METTL3-METTL14 proteins [78], is shown to reduce the half-life of IAP mRNAs by recruiting the m6A reader proteins YTHDF family (Figure 2), indicating that RNA methylation provides a protective effect in maintaining cellular integrity by clearing reactive ERVs-derived RNA species [79].

### 2.6. Exogenous Viruses Are Associated with ERVs Activation

Human ERVs activation can be triggered by infections of exogenous viruses such as HIV-1, hepatitis B virus (HBV), hepatitis C virus (HCV), human T-lymphotropic tumor virus-1 (HTLV-1), influenza A virus, and Kaposi’s Sarcoma-associated herpesvirus (KSHV) [80,81]. For HIV-1, the recombinant Tat protein upregulates HERV-K (HML-2) *gag* RNA transcripts in lymphocytes and monocytic cells through transcription factors NF-κB or NF-AT, indicating that exogenous viral infection activates transcription factors, which also bind to ERVs LTR regions and induce their activation [82]. An in-depth understanding of how ERVs are activated by exogenous viruses would facilitate the search for novel targets of virus-mediated diseases and therapeutic intervention.

### 2.7. Additional Factors in ERVs Transcriptional Regulation

Additional factors also contribute to transcriptional regulation of ERVs. TIP60, a lysine acetyltransferase, was found to be involved in silencing of ERVs, through positively regulating the expression of SUV39H1 and SETDB1, and thereby establishing global H3K9me3 levels [83]. KDM1A (also known as LSD1), a lysine-specific demethylase, was shown to be required to silence ERVs through regulating histone methylation and acetylation at LTR sequences. *Kdm1a* mutant mESCs exhibit increased methylation of histone H3K4, increased acetylation of H3K27, and decreased methylation of H3K9, indicating that chromatin modification mediated by KDM1A is part of the host’s defense against excessive ERVs activity [84]. Recently, the histone chaperone FACT, which is critical for nucleosome reorganization during replication, transcription, and DNA repair [85], was reported to recruit USP7 to repress MuERVL and MuERVL-fused 2C genes in mESCs by impeding the ubiquitination of H2Bub, providing insights into the regulation of TE-derived cryptic promoters during mammalian development and in diseases [86].

As a DNA-binding protein that is specifically expressed in two cell-stage embryos during mouse development [87], ZSCAN4C is positively associated with H3K27ac, H3K4me1 and H3K14ac deposition on MT2 (MuERVL LTR) and interacts with GBAF chromatin-remodeling complex to activate MT2 enhancer activity, indicating that ZSCAN4C plays a significant role in regulating MuERVL in mESCs [88]. *DUX4,* a eutherian-specific multicopy retrogene, encodes a transcription factor that can activate hundreds of retroviral elements (MuERVL/HERVL family) that define the cleavage-specific transcriptional programs in humans and mice [89]. In addition, it is shown that female sex hormones activate HERV-K through the OCT4 transcription factor in T47D breast cancer cells [90]. Notably, a recent study reported that TERT, the catalytic subunit of telomerase, can activate a subclass of ERVs independent of its telomerase activity to form double-stranded RNAs (dsRNAs), which trigger interferon signaling in cancer cells and promote an immunosuppressive tumor microenvironment [91]. 

## 3. Transcriptional Dysregulation of ERVs in Human Diseases

Several studies have suggested that TEs are domesticated for the benefit of the host. This process, in which the host makes use of TEs (including ERVs)-derived functions, are called exaptation, co-option, or repurposing [6]. Either *cis*-regulatory element activities or encoded proteins of ERVs can be beneficial to the host. For example, syncytin-1 and syncytin-2, which are specifically expressed in the placenta, are envelope proteins encoded by HERV-W and HERV-FRD, respectively, and with cell–cell fusogenic activities, contributing to the formation of placenta syncytiotrophoblast layer at the materno–fetal interface [92,93]. Therefore, capture of retroviral envelope genes may play a critical role in the emergence of placental mammals. Another example of exaptation is the *Fv1* gene of mice, which is an endogenous *gag* gene related to ERV-L family [94,95]. Fv1 confers host resistance to MuLV by blocking the incoming viral capsid cores shortly after entry [96,97]. Fv1 orthologues have been identified in a wide range of rodent species [98,99] and some Fv1 homologues restrict non-MuLV retroviruses [100], suggesting that Fv1 does not recognize conserved amino acid motifs but may instead detect structurally conserved spatial patterns in the hexameric lattice typical of retroviral capsid cores [97,101]. Notably, the neuronal Arc protein, which evolved from a Ty3/Gypsy retrotransposon Gag domain and has retained the topology of a retroviral Gag protein [102], is able to self-assemble into virus-like capsids that encapsulate RNA [103]. The Arc protein is released from neurons in extracellular vesicles and transfer the *Arc* mRNA into new target cells, where it can undergo activity-dependent translation, suggesting that Gag retroelements have been repurposed during evolution to mediate intercellular communication in the nervous system [103].

In spite of the exaptations of ERVs by the host, the dysregulation of them is involved in numbers of pathological processes. Although there is no direct evidence for ERVs causing diseases, aberrant expression profiles of the ERVs transcripts and their regulatory activities on proximal host genes have been identified in different diseases, such as cancer and neurodegenerative diseases. Mechanistically, ERVs may participate in pathological processes through several pathways: (i) ERVs act as promoters or enhance cellular gene expression through LTR *cis*-regulatory element activities; (ii) insertion of ERVs sequences induces chromosomal rearrangements and genome instability; (iii) ERVs encode proteins, long non-coding RNAs (lncRNAs), and double-stranded RNAs (dsRNAs) to affect host physiology.

### 3.1. HERVs in Cancer

The transcriptional activation of HERVs is a common feature in human cancers, suggesting that ERVs are causative elements or cofactors contributing to the onset and progression of human cancer [104]. So far, several studies have strongly suggested that ERVs play roles in various human cancers (Table 2).

Given the potential transposable ability of retrotransposon, it is the belief that the tumorigenicity of HERVs can depend on retroviral movement, thereby destabilizing the host genome [104]. Indeed, new insertions of TEs, especially ERVs, have been reported in several tumors [127]. LTRs can act as alternative promoter or enhancer, leading to the deregulation of proto-oncogenes or tumor suppressor genes [7]. A representative example is in Hodgkin’s lymphoma, where *CSF1R* transcription initiates at an LTR element of the MaLR THE1B family, rather than from its own promoter [123]. However, it should be noted that the LTRs activity may have an anti-oncogenic effect by driving the expression of tumor suppressor genes, such as *TP63* and *TNFRSF10B*, which are regulated by upstream LTRs belonging to the ERV9 group of HERVs [128,129].

HERVs can take a direct action via their own proteins in cancer. The envelop proteins of HERVs, such as syncytin-1 and HERV-K (HML-2) ENV, have been reported to contribute to tumorigenesis by inducing cell–cell fusion in melanoma [106], endometrial carcinoma [130], and breast cancer [121]. Furthermore, HERV-K (HML-2) ENV has also been shown to activate Ras/Raf/MEK/ERK and JNK/c-Jun signaling pathways, thereby promoting tumorigenesis and development [105,108,131], suggesting a direct interaction of ENV with cellular signaling pathways. Another mechanism by which ENV supports tumor progression is to promote immune escape by abolishing the anti-oncogenic cytolytic immune responses through its immunosuppressive domain (ISD) [132]. In addition to ENV, HERV proteins Rec and Np9 encoded by HERV-K (HML-2) are also regarded as tumor-specific biomarkers and act oncogenically by activating oncogene *c-MYC* or signaling pathways such as Notch, Wnt/β-catenin, Ras/ERK, and AKT [109,110,111,112,113]. 

lncRNAs play significant roles in various biological processes, including cancer progression. Strikingly, 75–83% of lncRNAs have been identified to contain TE sequences, especially ERVs [133]. Several HERVs-derived lncRNAs have been characterized in tumorigenesis and development. *UCA1*, a lncRNA consists of LTR7Y and HERV-H, has been shown to enhance proliferation, motility, invasion, and drug resistance of bladder cancer [114]. The HERVs-derived lncRNAs *SAMMSON* and *BANCR* are involved in melanoma progression [134,135], and linc-ROR contributes to progression, metastasis or chemoresistance in breast cancer [115], pancreatic cancer [116], and hepatocellular carcinoma [117]. A recent study identified a novel HCC (hepatocellular carcinoma)-specific lncRNA derived from MER52A, lncMER52A, which promotes invasion and metastasis of HCC cells by stabilizing p120-catenin [124]. Higher lncMER52A is associated with advanced TNM stage, less differentiated tumors, and shorter overall survival, and can serve as biomarker and therapeutic target for patients with HCC [124]. Another HERVs-derived lncRNA, EVADR, is revealed a striking association with adenocarcinomas, which are tumors of glandular origin, including colon, rectal, lung, pancreas, and stomach adenocarcinomas, and EVADR expression correlates with decreased patient survival [125]. Interestingly, a MER48 ERV element provides an active promoter to drive the specific activation of EVADR [125]. 

It has been reported that HERVs contribute to the modulation of innate immune response in different physiological and pathological conditions [104]. For example, lnc-EPAV, a full-length ERV-derived lncRNA, is a positive regulator of host innate immune responses by regulating expression of RELA, an NF-κB subunit that plays a critical role in antiviral responses [136]. Notably, dsRNAs derived from the bi-directional transcription of HERVs have opposite effects on modulating immune response in tumorigenesis and development. They may be involved in both anti-tumor defense and oncogenic process. On the one hand, dsRNAs from HERVs activated by DNMT inhibitors (DNMTis) in tumor cells can induce a growth-inhibiting immune response, and the high expression of genes associated with anti-viral response potentiates the response to immune checkpoint therapy [137,138]. On the other hand, HERVs-derived dsRNAs can also induce immune-suppressed microenvironment of tumors, similar to a chronic virally infected state [91,126]. These findings suggest significant implications of HERVs in cancer immunotherapy.

HERV deoxyuridine triphosphate nucleotidohydrolase (dUTPase) can trigger innate and adaptive immune responses [139]. In pulmonary arterial hypertension (PAH), the HERV-K dUTPase activates B cells, elevates cytokines in monocytes and pulmonary arterial endothelial cells, and increases pulmonary artery vulnerability to apoptosis, contributing to sustained inflammation and immune dysregulation [140]. Increased production and release of elastase, neutrophil extracellular traps, and vinculin-mediated increased adhesion in PAH are attributed to an increased in HERV-K dUTPase [141]. Another example of pro-inflammatory potential of HERV-K dUTPase is psoriasis, where HERV-K dUTPase proteins induce the activation of NF-κB through TLR2 to trigger the secretion of TH1 and TH17 cytokines involved in the formation of psoriatic plaques, supporting HERV-K dUTPase as a potential contributor to psoriasis pathophysiology [142]. Moreover, expression of dUTPase was identified in colorectal cancer and could be a predictive biomarker for the metastatic potential of colorectal cancer [143,144]. Interestingly, a recent study revealed that the expression of dUTPase determines whether elevation of the ribonucleotide reductase subunit R2 can lead to genome stress and chromosomal instability, and the combination of low dUTPase and high R2 in clinical tumor samples predicts poor survival in patients with colorectal cancer or breast cancers [145]. 

Considering the activation of HERVs in many human cancers and that HERVs expression has been shown to be associated with proliferation, metastasis, TNM stage, and overall survival, HERVs can be used as biomarkers for tumor diagnosis and/or prognosis [104]. For instance, a study reported that the combination testing of HERV-K (HML-2) with traditional prostate-specific antigen improves the efficacy of prostate cancer detection, specifically for older men and smokers who tend to develop a more aggressive disease [146]. HERVs have the potential to be targets for new cancer therapeutic opportunities as well. In this view, anti-HERV-K (HML-2) ENV antibodies have been shown to inhibit growth and induce apoptosis of breast cancer cells in vitro, and reduce growth of xenograft tumors in mice [147]. Consistently, HERV-K ENV-specific CAR^+^ T cells are able to lyse melanoma tumor cells in an antigen-specific manner [148]. Moreover, DNMTis activate the viral recognition and interferon response pathway by inducing dsRNAs transcribed by HERVs, which potentiates the response to immune checkpoint therapy [137,138]. 

### 3.2. HERVs in Aging and Neurodegenerative Diseases

As mentioned above, ERVs are largely transcriptionally silenced through heterochromatic structures. However, there may be a net loss of heterochromatin with aging, leading to the abnormal activation of TEs, including ERVs, in aging individuals [149,150]. It has been reported that IAPs and MusD are activated in aging mice [151,152]. In humans, HERV-K (HML-2) and HERV-W exhibit distinct expression patterns between young and old individuals [153]. Interestingly, the expression of HERV-H and HERV-W in peripheral blood mononuclear cells (PBMCs) was shown to be significantly positively correlated with age over 30 years [154]. Notably, HERV-W expression has been shown to significantly increase in individuals over 40 years old, and neurodegenerative diseases such as multiple sclerosis (MS) also occur in this age range [154]. Nowadays, ERVs have been implicated in the occurrence and development of neurodegenerative diseases, such as MS, amyotrophic lateral sclerosis (ALS), and autism spectrum disorder (ASD). 

MS is an autoimmune-mediated neurodegenerative disease of the central nervous system characterized by inflammatory demyelination with axonal transection [155]. Although the underlying etiology of MS is still not fully understood, the development of MS has been associated with activation of HERVs, especially HERV-W [4]. The presence of retroviral particles was first found in MS patients approximately 30 years ago [156,157] and subsequent studies revealed that these particles originated from HERV elements, originally called MS-associated retrovirus (MSRV), and now named HERV-W because it uses a tryptophan (W) tRNA as a primer for reverse transcription [158,159]. Mechanistically, HERV-W ENV can activate the innate immune system through a TLR4/CD14-dependent pathway and promote the development of a Th1 type of immune response upon DC activation [160]. HERV-W ENV-mediated activation of TLR4 leads to the induction of proinflammatory cytokines and inducible nitric oxide synthase, as well as the formation of nitrotyrosine groups and a subsequent reduction in myelin protein expression, resulting in an overall reduction of the oligodendroglial differentiation capacity and remyelination failure in MS [161]. Moreover, HERV-W ENV is also a potent superantigen associated with demyelination in MS, possibly related to molecular mimicry with myelin oligodendrocyte glycoprotein [162]. A recent study reported that HERV-W ENV induces a degenerative phenotype in microglial cells and drives them toward a close spatial association with myelinated axons, suggesting that HERV-W ENV-mediated microglial polarization contributes to neurodegeneration in MS [163]. Accordingly, treatment with neutralizing antibodies against HERV-W ENV abrogates the oligodendroglial maturation blockade [164]. In this view, the neutralizing antibodies have been used in a recently completed clinical study in MS patients, which showed that the antibody-mediated neutralization exerts neuroprotective effects [163]. In addition to HERV-W, other HERV elements have also been found in MS, such as HERV-H and HERV-K (HML-2) [165,166,167,168]. Taken together, these data suggest that activation of multiple HERVs families is linked to MS, among which HERV-W play a significant role.

Amyotrophic lateral sclerosis (ALS), a neurodegenerative disease characterized by progressive loss of cortical and spinal motor neurons, is another neurodegenerative disease associated with HERVs [162]. Activation of retroviral elements in ALS was first found through a study that identified RNA-directed DNA polymerase activity in brain tissue extracts from ALS patients, whereas no virus or transmissible agent was detected [169]. Subsequent studies confirmed the presence of reverse transcriptase in serum of ALS patients [170,171,172]; however, the attempts to search for exogenous retroviruses in ALS patients were unsuccessful [171,173], leading to the investigation of HERVs in ALS pathogenesis. As expected, a study revealed that HERV-K (HML-2) *pol* transcripts are upregulated in patients with ALS but not detectable in Parkinson disease or in healthy controls [174]. A subsequent study further identified the expression of HERV-K (HML-2) *pol*, *env*, and *gag* genes in brains of ALS patients [175]. Moreover, HERV-K (HML-2) ENV has also been detected in cortical and spinal neurons of ALS patients, but not in neurons from healthy individuals, which contributes to neurite retraction and beading, and neurodegeneration [175]. Several mechanisms by which HERV-K (HML-2) is activated in ALS have been revealed. For example, the nuclear translocation of IRF1 and NF-κB isoforms p50 and p65 has been revealed to contribute to the neuronal HERV-K (HML-2) activation in ALS brain tissue, implicating the critical role of neuroinflammation [176]. As a multifunctional protein dysregulated in ALS, *TDP-43* expression strongly correlates with HERV-K (HML-2) [174], and has been shown to activate HERV-K (HML-2) through binding to the LTR region of the provirus [175]. Besides HERV-K (HML-2), HERV-W ENV is also detected in muscle cells of ALS patients [177]. Nonetheless, although activation of HERVs is common in ALS, its pathogenic mechanisms require further investigation.

Recent studies have revealed aberrant expression of HERVs in neurodevelopmental disorder ASD. HERV-H is more abundantly expressed while HERV-W shows lower expression levels in PBMCs from ASD patients compared to healthy controls [178]. Furthermore, the expression of HERV-H is significantly upregulated in ASD patients with severe disease development [178]. Notably, HEMO, an ERV envelope protein of MER34 family [179], was reported to be altered in ASD patients and may be useful for the disease diagnosis [180]. Therefore, HERVs expression can be considered as a biomarker that is easily detectable in blood and may be helpful for early diagnosis of ASD. In addition, several studies have revealed that HERV-H, HERV-K (HML-2), HERV-L, and HERV-W are activated in Alzheimer’s disease (AD) [181,182,183]. In schizophrenia, several HERVs families have been shown to be dysregulated, including HERV-K (HML-2), HERV-W, ERV9, HERV-FRD, and HERV-H [184,185,186,187,188,189]. In addition, a recent study revealed that HERV-W ENV alters the NMDAR-mediated synaptic organization and plasticity through glia- and cytokine-dependent changes, leading to defective glutamate synapse maturation, behavioral impairments, and psychosis [190]. 

## 4. Conclusions and Perspective

ERVs are involved in various biological processes by encoding proteins, lncRNAs, dsRNAs, or acting as promoters/enhancers, thereby affecting human health and disease. Recent progress suggests that the implication of ERVs in cancer and neurodegenerative diseases provides an opportunity to develop novel therapeutic strategies. For example, nucleoside reverse transcriptase inhibitors (NRTIs) have shown promise in the treatment of neurodegenerative diseases [5]. DNMTis have been revealed to induce dsRNAs transcribed by ERVs in tumor cells, which activate the viral recognition and interferon response pathway, thereby enhancing the response to immune checkpoint therapy [137,138]. However, although many players in ERVs regulation have been identified, the detailed mechanisms of ERVs silencing and activation, especially the mechanisms of their action in human health and diseases, are not fully understood. Clearly, there are many species-, cell-type-, and disease-specific mechanisms, and unraveling which ERVs are silenced or activated, and how they are sensed in different contexts will be a major undertaking. The application of omics approaches, such as high-throughput sequencing, single-cell RNA-seq, genome editing technology, and proteomics can help to address these issues. Previously, ERVs have not received enough attention due to technical difficulties in analyzing these highly repetitive elements. With the development of technology, the mysteries of ERVs are being revealed step by step. However, the story of ERVs transcriptional regulation and the identification of specific ERV loci associated with specific diseases remains incomplete. Therefore, it is crucial and promising to enrich the knowledge of ERVs, our ancient “roommates” making up ~8% of human genome, in health and diseases.

## Figures and Tables

**Table 1:** Classification of ERVs.

ERVs Classes	Exogenous Counterpart	Representative ERVs
Class I	Gammaretrovirus Epsilonretrovirus	FeLV, GALV, KoRV, McERV, MDEV, MuERV-C, MuRRS, MuRVY, MuLV, GLN, VL30, PERV, **HERV-E, F, H, I, P, R, T, W, HERV-FRD**
Class II	Alpharetrovirus Betaretrovirus Deltaretrovirus Lentivirus	ALV, IAP, MMTV, MPMV, MusD/ETn, MINERVa, RELIK, **HERV-K (HML-1, 2, 3, 4, 5, 6, 7, 8, 9, 10)**
Class III	Spumaretrovirus	MuERV-L, **HERV-L**

Human ERVs are shown in bold. No Alpha-, Deltaretrovirus-, or Lentivirus-like elements are detectable in human genome.

**Table 2:** HERVs and oncogenic mechanisms in human cancers.

HERVs	HERVs Products/Activities	Oncogenic Mechanisms
HERV-K (HML-2)	ENV protein	ENV induces EMT and activates ERK pathway in breast cancer [105]. ENV mediates intercellular fusion in melanoma [106]. ENV maintains CD133+ melanoma cells with stemness features [107]. ENV promotes pancreatic cancer proliferation, tumorigenesis, and metastasis by activating RAS/MEK/ERK and JNK/c-Jun signaling pathways [108].
	Rec protein	Rec activates *c-MYC* by overcoming the transcriptional repression of testicular zinc-finger protein (TZFP) for *c-MYC* promoter [109]. Rec relieves the repression of androgen receptor (AR) activity by forming a trimeric complex with TZFP and AR [109], or binds to the human small glutamine-rich tetratricopeptide repeat protein (hSGT) [110].
	Np9 protein	Np9 interacts with ligand of Numb protein X, affecting tumorigenesis through the LNX/Numb/Notch pathway [111]. Np9 as a critical molecular switch of multiple signaling pathways in leukemia [112]. Rec and Np9 derepressed *c-MYC* through the inhibition of promyelocytic leukemia zinc-finger protein (PLZF) [113].
HERV-H	lncRNA	*UCA1* enhances proliferation, motility, invasion, and drug resistance of bladder cancer [114]. linc-ROR contributes to progression, metastasis, or chemoresistance in breast cancer [115], pancreatic cancer [116], and hepatocellular carcinoma [117].
	*cis*-regulatory element	LTR acts as alternative promoter for *GSDML* in cervical cancer [118,119].
HERV-E	*cis*-regulatory element	Modulating *PLA2G4A* transcription in urothelial cancer [120].
syncytin-1/ERVW-1	ENV protein	Syncytin-1 mediates cancer–endothelial cell fusions in breast cancer [121].
ERV-9	lncRNA	*PRLH1* plays an important role in the formation of RNA–protein complex that promotes the HR-mediated DSB repair [122].
MaLR	*cis*-regulatory element	LTR acts as alternative promoter for *CSF1R* in Hodgkin’s lymphoma [123].
MER52A	lncRNA	lncMER52A promotes invasion and metastasis of hepatocellular carcinoma cells by stabilizing p120-catenin [124].
MER48	lncRNA/*cis*-regulatory element	lncRNA EVADR is associated with adenocarcinomas, and a MER48 ERV element acts as an active promoter for its specific activation [125].
Multiple HERVs	dsRNA	dsRNAs derived from the bi-directional transcription of HERVs induce an immunosuppressive tumor microenvironment [91,126].

## Data Availability

Not applicable.
